# Autogenous bone graft collection by acetabular reamers at the ilium yielded comparable volume with less blood loss and lower costs compared to the Reamer–Irrigator–Aspirator 2 system at the femur

**DOI:** 10.1007/s00590-025-04347-9

**Published:** 2025-06-13

**Authors:** Andrew Duong, Vishal Patel, Soroush Shabani, Vivek Satish, Michael Allen, Ryan Ross, Ashley Mulakaluri, Joseph Patterson

**Affiliations:** 1 University of Southern California, Los Angeles, United States; 2 University of California San Francisco, Fresno, United States

**Keywords:** Bone graft, Autograft, Pelvis, Femur, Nonuion, Reamer irrigator aspirator

## Abstract

**Objective:**

To compare autogenous bone graft volume, blood loss, transfusion rate, complications, and costs by collection using acetabular reamers at the ilium (ARI) to Reamer–Irrigator–Aspirator (RIA2) at the femur.

**Materials and methods:**

Adults who underwent long bone or sacral nonunion repair with autogenous bone graft collection by either unilateral ARI or RIA2 from femur from November 2020 to May 2023 at two academic trauma referral centers were retrospectively identified. Outcomes included graft volume, estimated blood less (EBL), perioperative change in hematocrit (ΔHct) and hemoglobin (ΔHgb), red blood cells (pRBC) units transfused intra- or postoperatively, infection, iatrogenic fracture, pathologic fracture, venous thromboembolism, and equipment costs per procedure.

**Results:**

Twenty-nine patients were included, of whom 18 received ARI was and 11 received RIA2. No differences were observed between groups regarding age, sex, body mass index, substance use, or medical comorbidities. Mean graft volumes were similar: ARI 38.3 cc (range 20–80 cc) versus RIA2 36.4 cc (range 10–60 cc), *p* = 0.74). ARI was associated with lower mean EBL (408.7 ± 210.0 cc vs. 750.0 ± 508.0 cc, *p* = 0.02), mean ΔHgb (2.1 ± 1.4 g/dL vs. 4.0 ± 1.7 g/dL, p = 0.007) and mean ΔHct (6.9 ± 4.9% vs. 11.9 ± 5.7%, *p* = 0.03). No differences in the incidence of pRBC transfusions (0.28 ± 0.67 vs. 0.64 ± 1.03 units, *p* = 0.32) or complications at the harvest site (5.6% vs. 0%, *p* = 1.00) were observed. ARI durable components acquisition ($11,500 vs. $12,500) and per case sterilization and disposable ($18 vs. $3,500) costs were lower.

**Conclusions:**

Unilateral autogenous bone graft collection from ilium with acetabular reamers yielded similar graft volume with less blood loss at lower cost than RIA2 collection from femur.

**Supplementary Information:**

The online version contains supplementary material available at 10.1007/s00590-025-04347-9.

## Introduction

Globally, over two million bone graft procedures are performed each year [1, 2]. Bone graft is frequently used for treating sequelae of skeletal trauma including nonunion, post-traumatic arthritis, and critical-sized bone defects [3, 4]. Autogenous bone graft (autograft) provides osteoconductive, osteoinductive, and osteogenic properties, confers minimal risk of immunogenic rejection, and may provide structural support depending on how the graft is harvested [1]. These characteristics make autogenous bone graft an attractive adjunct in the repair of fracture nonunion and critical-sized defects of the pelvis and long bones. Large volume defects in weight-bearing portions of the appendicular skeleton require large volumes of bone graft, which requires morbid collection procedures often at one or more additional surgical sites, with additional surgical time, surgical blood loss, and risk of infection as well as risks unique to the collection method and donor site [5–7]. Collection methods for large volumes of autogenous bone graft must maximize yield while minimizing morbidity and expense.

The ilium and femur are potential sources, among many, of large volumes autograft bone for post-traumatic skeletal reconstruction surgeries. Dick described the off-label use of acetabular reamers to procure a bone paste from the outer surface of the ilium [8]. Reaming the outer table of the gluteal pillar or posterior ilium with a hemispherical reamer such as those designed for the acetabulum (Acetabular Reamers to Ilium; ARI) quickly provides 20–40 cubic centimeters (cc) of corticocancellous autograft with a low rate of major complications such as pelvic fracture or deep infection but moderate rate of minor complications including pain at donor site, neuralgia of the lateral femoral cutaneous nerve, and superficial infection [9–12]. Alternatively, 25–90 cc of corticocancellous autograft may also be collected from the endosteum of the femur with a Reamer–Irrigator–Aspirator (RIA2) device [13, 14]. The RIA2 offers a less invasive approach and similar quality graft but applies a continuous negative pressure force which may incur greater surgical blood losses and transfusion risk, and may also be associated with greater equipment costs [6, 12, 15]. While alternate sources of autogenous bone including the tibial shaft, proximal tibia, distal femur, trochanteric region, posterior iliac crest, ribs, proximal humerus and distal radius are available, the iliac crest and femur have generally been reported to yield the greatest volumes of graft with a single collection effort [5–7].

The purpose of this investigation was to compare autogenous bone graft collection method using ARI to RIA2 at the femur by graft volume, blood loss, intra- or postoperative red blood cell transfusions, complications at the graft harvest site, and costs attributable to equipment and materials. The hypothesis was that ARI would be associated with less blood loss, transfusion risk, and equipment and materials costs, but would not be associated with significantly different graft volume with complications at the graft harvest site compared to RIA2.

## Patients and methods

### Study sample selection

A retrospective cohort study was conducted at one Level 1 trauma center and one academic medical center. Patients age ≥ 18 years who underwent surgery related to a long bone or sacral fracture nonunion from November 2020 to May 2023 by one fellowship-trained orthopedic trauma surgeon were identified from surgical case logs using common procedural terminology codes for nonunion repair procedures. Patients with missing data were excluded. Treatment was classified by autologous bone graft collected from the femur by RIA2 device or ARI. The choice of treatment was at the discretion of the treating surgeon and was influenced by the presence of medullary implants within the pelvis and/or femurs. Total hip arthroplasty, obesity, and prior pelvic or abdominal surgery was not considered contraindications to the use of ARI. Patients were excluded if bone graft was not collected using either technique, if both techniques were used, or if preoperative and postoperative hemoglobin or hematocrit values were not available.

### Technique description

ARI bone autograft harvests were performed per the method described by Westrich et al. [11]. Briefly, an 8–12 cm incision is made 2 cm distal and parallel to the iliac crest 2 cm posterior to the anterior superior iliac spine. This is somewhat caudal and parallel to the typical lateral window incision for exposure of an acetabular fracture. The outer table of the ilium is then exposed in a supraperiosteal plane with a Cobb elevator and laparotomy sponge, elevating the abductor musculature with a cuff of tendinous origin and protecting this muscle sleeve with a malleable retractor distal. Beginning with a 36 mm diameter acetabular reamer (Zimmer Biomet, Warsaw, IN), sequentially larger diameter acetabular reamers are applied to the outer table of the ilium centered over the gluteal pillar using a high-torque, low-speed reaming technique. Once the outer cortex is breached, successively reamers are used to dilate the collection site defect diameter without violating the inner table of the ilium or compromising the cranial cortical contour of the iliac crest. In this manner, the subcutaneous bony contour of the pelvis is preserved. When no larger reamer may be safely applied, additional graft is collected anteriorly, posteriorly, cranially, and distally with angled curettes. The wound is irrigated and closure is performed by approximation of the fascial origins of the gluteus medius and tensor fascia latae to the insertion of the external abdominal oblique on the iliac crest. A video of the ARI technique is provided as Supplementary Material. While the unmodified lateral window may be used for this purpose in association with acetabular fracture repair, consideration should be given to devascularization of the ilium by exposure of the inner and outer tables over a large area.

Femoral autograft was collected by antegrade or retrograde application of the RIA2 device (J&J MedTech, DePuy Synthes, Warsaw, IN) to the femoral medullary canal over a guidewire. The size of the first streamer head was assessed based on preoperative radiographs of the femur as well as the fluoroscopic appearance you have the diameter of the femoral canal relative to the diameter of the 12 mm cannulated drill bit or 12 mm cannulated awl used to perforate the femur prior to guidewire placement. Collection was then performed using reamer head(s) of diameter(s) sized from preoperative radiographs of the femur until satisfactory graft volume and quality was obtained [13].

Graft volume with both techniques was measured by collecting the graft in a graduated specimen container, decanting effluent and blood separately to any allograft if planned for use, then estimating the volume based on the graduated markings.

### Data collection

Demographic, injury, treatment characteristics, and outcomes including autograft volume, surgical blood loss estimated by the surgeon, transfusions, and postoperative complications were abstracted from electronic medical records and operative reports. Demographic data included age, sex, body mass index (BMI), tobacco use, substance use, and Charlson Comorbidity Index (CCI). Injury characteristics included nonunion location, etiology determined by callus appearance on plain radiograph as described by Panagiotis, and septic/aseptic classification by operative cultures [16]. Cases with concern for possible infection were treated in a staged manner with bone biopsy and antibiotic spacer exchange until negative cultures were obtained before proceeding with nonunion repair.

Treatment data included allograft use, allograft volume (cc), autograft harvesting method, the use of prophylactic internal fixation. Outcomes included bone autograft volume collected (cc), surgical blood loss estimated by the surgeon, difference between preoperative and postoperative hematocrit (Hct) and hemoglobin (Hgb), and units of red blood cells (pRBC) transfused intra- and postoperatively. Postoperative complications included venous thromboembolism as well as complications at the graft collection site included superficial surgical site infection requiring oral antibiotics, deep surgical site infection requiring surgical debridement, intraoperative iatrogenic fracture, postoperative pathologic, and reoperation for complications related to the bone graft collection. Costs attributable to equipment and materials were ascertained from itemized patient charges and institution-specific sterile processing charges.

### Statistical analysis

Categorical variables were compared between RIA2 and ARI procedures using Fisher’s exact test. Continuous variables were compared between groups by student’s *t-*test. A *p* value of < 0.05 was considered significant. Statistical tests were performed using R statistical software (R Foundation, Vienna, Austria). This investigation was not funded.

## Results

**Fig. 1:**
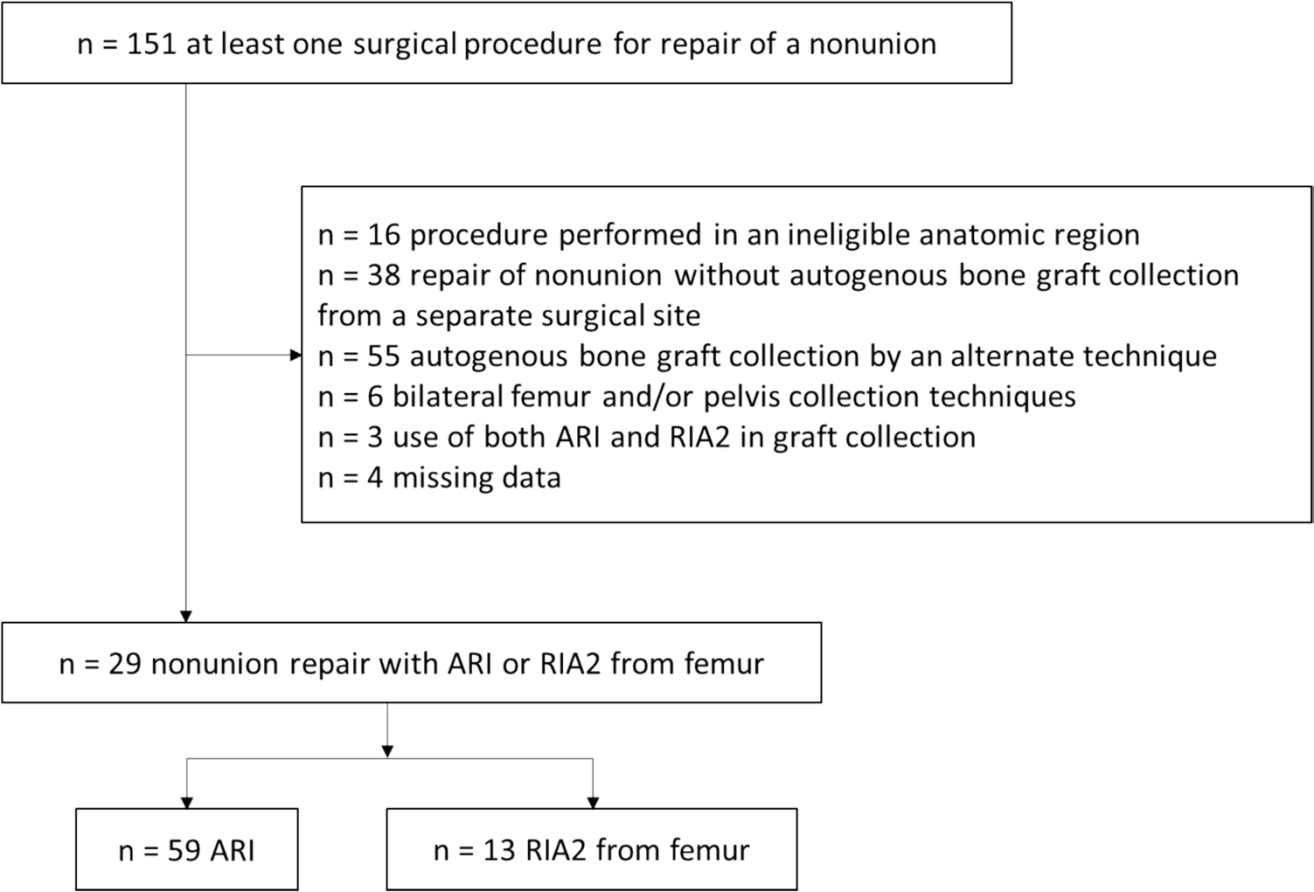
Study sample selection flowchart

The screened population included 151 patients who received at least one surgical procedure for repair of a nonunion identified from case logs. One hundred twenty-two patients were excluded (Fig. 1). The study sample included twenty-nine with a mean age of 53.0 years (range 20–86), 79.3% male, BMI 30.3 ± 8.6 kg/m^2^, and CCI 2.1 ± 2.2. Sixteen femora, 10 tibiae, 2 sacra, and 1 humerus were treated. Nonunion etiologies were 9 septic, 16 oligotrophic, 2 atrophic and 2 hypertrophic.

Eighteen (62.1%) patients underwent nonunion repair with ARI autogenous bone graft collection and 11 (37.9%) patients were treated with RIA2. No differences in age, sex, substance use, CCI, nonunion type or location were observed between treatment groups (Table 1). The mean volume of autogenous bone graft collected for the entire cohort was 37.6 cc (range 10–80 cc). No difference was observed in the volume of graft collected by ARI (mean 38.3 cc, range 20–80 cc) versus RIA2 (mean 36.4 cc, range 10–60 cc; *p* = 0.74). No differences in the frequency or volume of supplemental allograft or use of prophylactic stabilization of the harvest site were observed between collection methods (Table 2). Stabilization of the harvest site (*p* = 1.00) between groups (Table 2).

**Table 1:** Demographic and nonunion characteristics

Demographic	Total Cohort (n = 29)	ARI (n = 18)	RIA2 (n = 11)	*p* value
Mean Age, Years	53.0 (SD = 17.8)	53.4 (SD = 19.5)	52.3 (SD = 15.7)	0.96
Sex, Male	23 (79.3%)	14 (77.8%)	9 (81.8%)	1.00
BMI (kg/m^2^)	30.3 (SD = 8.6)	30.2 (SD = 8.6)	30.5 (SD = 8.9)	0.93
Tobacco use	3 (10.3%)	3 (16.7%)	0 (0.0%)	0.25
Substance use	4 (13.8%)	2 (11.1%)	2 (18.2%)	0.62
Mean Charlson Comorbidity Index	2.1 (SD = 2.2)	2.1 (SD = 2.4)	2.2 (SD = 2.2)	0.84
Nonunion type Septic Oligotrophic Atrophic Hypertrophic	9 (31.0%) 16 (55.2%) 2 (6.9%) 2 (6.9%)	3 (16.7%) 12 (66.7%) 1 (5.6%) 2 (11.1%)	6 (54.5%) 4 (36.3%) 1 (9.1%) 0 (0.0%)	0.13
Injury Site Femur Tibia Humerus Sacrum	16 (55.2%) 10 (34.5%) 1 (3.4%) 2 (6.9%)	11 (61.1%) 4 (22.2%) 1 (5.6%) 2 (11.1%)	5 (45.5%) 6 (54.5%) 0 (0.0%) 0 (0.0%)	0.24

ARI: acetabular reamers to ilium. RIA2: Reamer–Irrigator–Aspirator 2 device to femur. BMI: body mass index

**Table 2:** Treatment and outcomes

Outcome	Total Cohort (n = 29)	ARI (n = 18)	RIA2 (n = 11)	*p* value
Autograft Volume (cc)	37.6 (SD = 15.2)	38.3 (SD = 15.3)	36.4 (SD = 15.5)	0.74
Allograft Use	14 (48.3%)	9 (50.0%)	5 (45.5%)	1.00
Allograft Volume (cc)	21.2 (SD = 25.2)	27.1 (SD = 27.6)	13.6 (SD = 20.6)	0.19
Prophylactic Internal Fixation	2 (6.8%)	1 (5.6%)	1(9.1%)	1.00
Operative Time, Minutes	318.0 (SD = 138.3)	307.5 (SD = 127.2)	335.2 (SD = 166.5)	0.64
Preoperative Hgb	12.5 (SD = 2.1)	12.3 (SD = 2.3)	12.8 (SD = 1.9)	0.54
Postoperative Hgb	9.7 (SD = 1.6)	10.2 (SD = 1.5)	8.9 (SD = 1.6)	0.03*
ΔHgb	2.8 (SD = 1.8)	2.1 (SD = 1.4)	4.0 (SD = 1.7)	0.007*
Preoperative Hct (%)	39.4 (SD = 5.6)	39.5 (SD = 4.9)	39.1 (SD = 6.9)	0.87
Postoperative Hct (%)	30.6 (SD = 4.7)	32.6 (SD = 3.7)	27.3 (SD = 4.4)	0.003*
ΔHct	8.8 (SD = 5.7)	6.9 (SD = 4.9)	11.9 (SD = 5.7)	0.03*
Estimated Blood Loss	537.9 (SD = 384.0)	408.7 (SD = 210.0)	750.0 (SD = 508.0)	0.02*
Perioperative Blood Transfusion	7 (24.1%)	3 (16.7%)	4 (36.4%)	0.37
Units of Red Blood Cells Transfused	0.41 (SD = 0.82)	0.28 (SD = 0.67)	0.64 (SD = 1.03)	0.32
Any Complication	1 (3.4%)	1 (5.6%)	0 (0.0%)	0.62
Superficial SSI	1 (3.4%)	17	0 (0.0%)	1.00
Deep SSI	0 (0.0%)	0 (0.0%)	0 (0.0%)	–
Iatrogenic Fracture at Harvest Site	0 (0.0%)	0 (0.0%)	0 (0.0%)	–
Pathologic Postoperative Fracture at Harvest Site	0 (0.0%)	0 (0.0%)	0 (0.0%)	–
Venous Thromboembolism	0 (0.0%)	0 (0.0%)	0 (0.0%)	–

Values given as means. ARI: acetabular reamers to ilium. RIA2: Reamer–Irrigator–Aspirator 2 device to femur. SD: Standard deviation. Hbg: hemoglobin. Hct: hematocrit. SSI: surgical site infection

*statistically significant values

Observed and estimated blood loss were greater in patients treated with autograft harvested by RIA2. Preoperative Hgb (12.3 ± 2.3 g/dL vs. 12.8 ± 1.9 g/dL, *p* = 0.54) and Hct (39.5 ± 4.9% vs. 39.1 ± 6.9%, *p* = 0.87) were not different between groups. RIA2 harvest was associated with significantly lower postoperative Hgb (10.2 ± 1.5 g/dL vs. 8.9 ± 1.6 g/dL, *p* = 0.03) and Hct (32.6 ± 3.7% vs. 27.3 ± 4.4%, *p* = 0.003) as well as significantly greater ΔHgb (2.1 ± 1.4 g/dL vs. 4.0 ± 1.7 g/dL, *p* = 0.007) and ΔHct (6.9 ± 4.9% vs. 11.9 ± 5.7%, *p* = 0.03) compared to ARI. Estimated blood loss was lower in the ARI group (408.7 ± 210.0 cc vs. 750.0 ± 508.0 cc, *p* = 0.02). No significant differences were observed in the frequency of pRBC transfusion 17.6% vs. 36.4%, *p* = 0.32) nor number of pRBC units transfused ((0.28 ± 0.67 units vs. 0.64 ± 1.03 units, *p* = 0.32).

One patient in the ARI group (5.6%) developed a superficial infection at the bone graft collection site, characterized as staple erythema and serous wound drainage. This resolved with staple removal, holding anticoagulation medication for 5 days, and 5 days of oral cephalexin 500 mg QID. No patient experienced a deep SSI, iatrogenic nerve injury, iatrogenic intraoperative fracture, or postoperative pathologic fracture at the harvest site, or venous thromboembolism were observed between groups Table 2). No reoperation or change in postoperative plan such as weight-bearing restrictions related to bone graft collection occurred in either group.

The costs of durable components acquisition of the RIA2 system were $12,500 once with per-case disposable costs of approximately $3500 and no planned maintenance costs. Conversely, the acquisition costs for ARI were $11,500 once with per case-cast costs of $18 for consigned equipment sterilization with $1800 in maintenance costs including reamer sharpening services every three years.

## Discussion

In this multicenter retrospective cohort of 29 patients with long bone or sacral nonunion, harvesting autogenous bone graft from the outer aspect of the ilium with an acetabular reamer for nonunion repair yielded similar graft volume as RIA2 autograft harvest from the femur with lower estimated and objective blood losses and lower estimated procedure expense, and no significant difference in intraoperative time. One superficial surgical site infection was observed with the ARI technique and resolved without additional cervical intervention. These findings represent a novel direct comparison of the ARI technique of ilium bone graft harvest to the RIA2 procedure.

Autograft bone is a common adjunct for nonunion repair. Surgeons may elect to treat larger defects and atrophic nonunions with greater volume of autograft. Prior reports suggest that RIA devices may yield larger graft volume (25–90 cc) compared to series of harvests obtained from the anterior iliac crest (5–72 cc) and similar volume to reaming the posterior iliac crest (25–88 cc) [7, 14, 17]. In our experience, clinically and statistically equivalent volumes were harvested via RIA2 from the femur (36.3 cc) compared to ARI (37.9 cc). The volume harvested with RIA2 in this series is lower than prior reports and may reflect a conservative harvest technique. Prophylactic fixation of the femur after RIA2 harvest was performed in one morbidly obese patient and one patient with poor bone quality, and no pathologic or iatrogenic femur fracture. The present findings are also consistent with a meta-analysis performed by Oliva et al., which demonstrated no difference in volume between RIA2 and all manner of iliac crest bone graft harvest methods [6, 17].

Clinically relevant blood loss is described after autograft harvest with RIA devices. The closed suction system is direct connection with the peripheral venous and arterial systems of the lower extremity via the richly vascular femoral medullary canal and endosteum, actively exsanguinates the patient with aspiration of reamed endosteum [14]. Laubach et al., in a meta-analysis examining complications after RIA use, reported EBL of 803.3 cc; by comparison, similar to the 750 cc observed in this study [18]. EBL following harvest from the ilium is lower, ranging from 232 to 474 cc [7, 18]. Scharfenberger and Weber reported ΔHgb and ΔHct postoperatively at 4.3 g/dL and 11%, respectively, after RIA. Comparatively, Ahlmann et al. observed lower ΔHct of 4.9% and 3.6% after graft harvest from the posterior and anterior iliac crest, respectively [19]. Marchand et al. found that bone harvest via RIA was significantly associated with a greater Hct drop than from iliac crest bone graft and greater odds of transfusion after RIA compared to traditional iliac crest harvest methods, while Oliva et al. found no difference between the RIA and iliac crest regarding transfusion risk [6, 14]. In the present study, EBL, ΔHgb, and ΔHct were comparable to these previous investigations, supporting our conclusion that ARI incurs less blood loss than RIA2 from the femur. Of note, supplemental allograft was used more frequently in ARI cases despite equivalent autograft volumes, indicating larger volume nonunion defects. Procedures for larger volume ostensibly require greater exposure and may be associated with greater blood loss not attributable to the bone graft harvest, biasing comparisons of EBL in favor of RIA2. Despite this, ΔHgb and ΔHct were both greater in the RIA2 group.

Potential complications of ARI and RIA2 bone graft harvest are relatively specific to each technique. Comparing complication risk between these techniques is somewhat difficult due to heterogeneity in prior definitions. Migliorini et al. described an 8.6% incidence of complications after procurement from the iliac crest, with chronic pain, lateral femoral cutaneous nerve lesions, and iliac wing fractures being the most common [9]. Bimmel and Govaers, described in their retrospective series of 78 patients undergoing iliac crest harvest with an acetabular reamer, a 25.6% rate of postoperative paresthesia in a specific nerve distribution [12]. Conversely, Brawley and Simpson described a series of 34 patients experiencing no postoperative complications after harvest with acetabular reamer from the iliac crest, including infection, hematoma, paresthesia, or persistent donor site pain [20]. In systematic reviews, Laubach et al. reported a total complication rate of 1.7% after procurement by the predecessor RIA system, while Oliva et al. reported RIA was associated with lower odds of harvest site pain, infection, or any adverse event compared to traditional iliac crest harvest. In contrast, we observed no difference in infection or overall complication rate between RIA2 and ARI bone graft harvest. Although there was an insignificantly higher rate of wound complication within the ARI group, most of these ARI infections were at the operative nonunion site rather than graft harvest location. Additionally, patient factors such as comorbidities and a history of infection at the operative site likely predisposed these patients to post-surgical complications likely unrelated to the graft harvest method. Anecdotally, patients receiving the ARI describe minimal pain at the harvest site by the first postoperative visit for a wound check at 2–3 weeks.

Equipment acquisition costs, maintenance costs, and per-case disposable costs were favorable with ARI. Dawson et al. performed a cost analysis favoring RIA over iliac crest for large volume bone graft harvest even though RIA costs approximately $600 USD more [17]. Nodzo et al. estimated the cost of a RIA system to be $998, suggesting that the higher price may be offset by decreased patient morbidity after harvest.[21]. Adjusted for inflation, our estimate is comparable to previously reported figures for the older-generation RIA system of $968–$1310 [17, 21, 22]. Our data indicate that ARI may be more cost-effective than RIA2, as it yields similar volume of bone graft with similar up-front costs and lower per-use costs.

This study has limitations including those inherent to a retrospective investigation of a single surgeon’s experience, including bias due to patient selection, treatment allocation, censorship due to differential follow-up, and generalizability to other patients, surgeons, and practice environments. Nonunion repair surgeries are uncommon and heterogenous, and the treatment alternatives described are not identical case-to-case, which limit the validity of comparisons and prevents inference of casual effects of treatments on outcomes. Patients who received ARI may have had prior ipsilateral and/or contralateral femur surgery precluding the use of RIA2 or limiting the volume of graft collected due to previous instrumentation. The small sample size of 29 patients did not provide sufficient statistical power required to detect clinically relevant differences in outcomes such as bone graft volume collection, transfusion rate, or complications. While we investigated rates of infection and iatrogenic fracture, we did not systematically collect specific measures of harvest site pain or nerve injury. Nonetheless, autograft bone harvests were performed consistently and routinely by a surgeon in two distinct practice environments.

## Conclusion

Unilateral autogenous bone graft collection from the ilium with acetabular reamers for repair of nonunion yielded similar graft volume with less blood loss at lower cost than RIA2 collection from the femur. Complications with either technique were rare, and no patient required additional surgical intervention or a change in postoperative care. Prospective study of the safety and efficacy of bone graft collection by acetabular reamers versus RIA2 for nonunion repair, perhaps with randomization of treatments, is warranted.

## Supplementary Information

Below is the link to the electronic supplementary material.

Supplementary file1 (MP4 27094 KB)

## Data Availability

No datasets were generated or analyzed during the current study.
